# Clinical and genetic characteristics of 42 Chinese paediatric patients with X-linked adrenal hypoplasia congenita

**DOI:** 10.1186/s13023-023-02737-y

**Published:** 2023-05-26

**Authors:** Wanqi Zheng, Ying Duan, Yu Xia, Lili Liang, Zhuwen Gong, Ruifang Wang, Deyun Lu, Kaichuang Zhang, Yi Yang, Yuning Sun, Huiwen Zhang, Lianshu Han, Zizhen Gong, Bing Xiao, Wenjuan Qiu

**Affiliations:** Department of Paediatric Endocrinology and Genetic Metabolism, Xinhua Hospital, Shanghai Institute of Paediatric Research, School of Medicine, Shanghai Jiao Tong University, 1665 Kongjiang Road, Shanghai, 200092 China

**Keywords:** Adrenal hypoplasia congenita, Hypogonadotropic hypogonadism, DAX1, NR0B1, Children, Variant

## Abstract

**Background:**

X-linked adrenal hypoplasia congenita (AHC) is a rare disorder characterized by primary adrenal insufficiency (PAI) and hypogonadotropic hypogonadism (HH), with limited clinical and genetic characterization.

**Methods:**

The clinical, biochemical, genetic, therapeutic, and follow-up data of 42 patients diagnosed with X-linked AHC were retrospectively analysed.

**Results:**

Hyperpigmentation (38/42, 90%), vomiting/diarrhoea (20/42, 48%), failure to thrive (13/42, 31%), and convulsions (7/42, 17%) were the most common symptoms of X-linked AHC at onset. Increased adrenocorticotropic hormone (ACTH) (42/42, 100%) and decreased cortisol (37/42, 88%) were the most common laboratory findings, followed by hyponatremia (32/42, 76%) and hyperkalaemia (29/42, 69%). Thirty-one patients presented with PAI within the first year of life, and 11 presented after three years of age. Three of the thirteen patients over the age of 14 exhibited spontaneous pubertal development, and ten of them experienced delayed puberty due to HH. Six patients receiving human chorionic gonadotropin (hCG) therapy exhibited a slight increase in testicular size and had rising testosterone levels (both *P* < 0.05). The testicular volumes of the three patients with pulsatile gonadotropin-releasing hormone (GnRH) therapy were larger than those of the six patients undergoing hCG therapy (*P* < 0.05), and they also exhibited some growth in terms of luteinizing hormone (LH), follicle-stimulating hormone (FSH), and testosterone. Of the 42 patients, three had an Xp21 deletion, and 39 had an isolated DAX1 defect. Most patients (9/10) with entire *DAX1* deletion accounting for 23.8% (10/42) of the total variants had early onset age of less than one year.

**Conclusions:**

This study details the clinical features and genetic spectra of X-linked AHC. Patients with X-linked AHC show a bimodal distribution of the age of onset, with approximately 70% presenting within the first year of life. Pulsatile GnRH may be recommended for HH when hCG therapy is not satisfactory, although it is difficult to achieve normal testicular volume. The combination of clinical features and molecular tests provides information for an accurate diagnosis.

## Introduction

X-linked adrenal hypoplasia congenita (AHC, OMIM 300200, ORPHA: 95702) is a potentially life-threatening disorder caused by defects in *DAX1* (dosage-sensitive sex reversal-adrenal hypoplasia congenita critical region on X chromosome 1, gene 1), which is also known as *NR0B1* (nuclear receptor subfamily 0, Group B, member 1; OMIM 300473), with a prevalence of one in 70,000 to 600,000 males [1–3]. DAX1 is located on chromosome Xp21.2 and has two exons that encode a 470-amino-acid protein belonging to the nuclear receptor superfamily. The structure of DAX1 consists of an amino-terminal DNA binding domain (DBD) containing 3.5 repeated motifs and a carboxyl-terminal ligand-binding domain (LBD) with twelve helices [4–6]. The exact biological mechanism of DAX1 remains unclear. Most studies have shown that DAX1 expressed in steroidogenic tissues (adrenals and gonads), the hypothalamus, and the pituitary is a transcriptional repressor that unexpectedly plays a positive role in the steroidogenic pathway and the development of the hypothalamic-pituitary–gonadal (HPG) axis. Loss of function of DAX1 results in adrenal hypoplasia and reproductive dysfunction [5, 7–9].

Patients with X-linked AHC usually present with signs and symptoms of primary adrenal insufficiency (PAI) in infancy or early childhood, including hyperpigmentation, vomiting, diarrhoea, failure to thrive, and convulsions. Biochemical investigations include increased adrenocorticotropic hormone (ACTH), decreased cortisol, hyponatremia, and hyperkalaemia [3, 7, 10, 11]. Meanwhile, patients in adolescence generally manifest with delayed puberty owing to hypogonadotropic hypogonadism (HH) and men exhibit impaired fertility due to azoospermia [12–16]. DAX1 defects in the X-linked AHC can range from point variants to isolated deletions involving parts of or the entire *DAX1* gene up to Xp21 deletions. Xp21 deletion usually contains *DAX1* (resulting in X-linked AHC), *GK* (resulting in glycerol kinase deficiency), and *DMD* deletion (resulting in Duchenne muscular dystrophy). Intellectual disability (ID) has been reported in males with Xp21 deletion syndrome (OMIM 300679, ORPHA: 261476) when the deletion involves *IL1RAPL1* or extends distally to contain *DMD* [3, 10, 17].

Pubertal development in affected boys with DAX1 defects is a matter of concern since most boys with HH experience delayed puberty, while a small percentage of patients experience disruption when entering puberty naturally [18, 19]. Pulsatile gonadotropin-releasing hormone (GnRH), gonadotropin medication [human chorionic gonadotropin (hCG) alone or coupled with follicle-stimulating hormone (FSH)], or testosterone replacement can all be used to virilize adolescents for the treatment of DAX1-related HH. To date, published studies on HH related to DAX1 defects are primarily case reports [11, 13–15, 20–24]. The standard regimen to date remains controversial, and experience with therapeutic effects is limited.

In the present study, to gain a comprehensive understanding of X-linked AHC, we report the DAX1 defect of 42 Chinese patients with X-linked AHC and review the clinical and laboratory characteristics of adrenal insufficiency or a combination with Xp21 deletion syndrome. In addition, we retrospectively analyse the data for features of pubertal development and treatment evaluation for HH.

## Patients and methods

### Patients

**Fig. 1:**
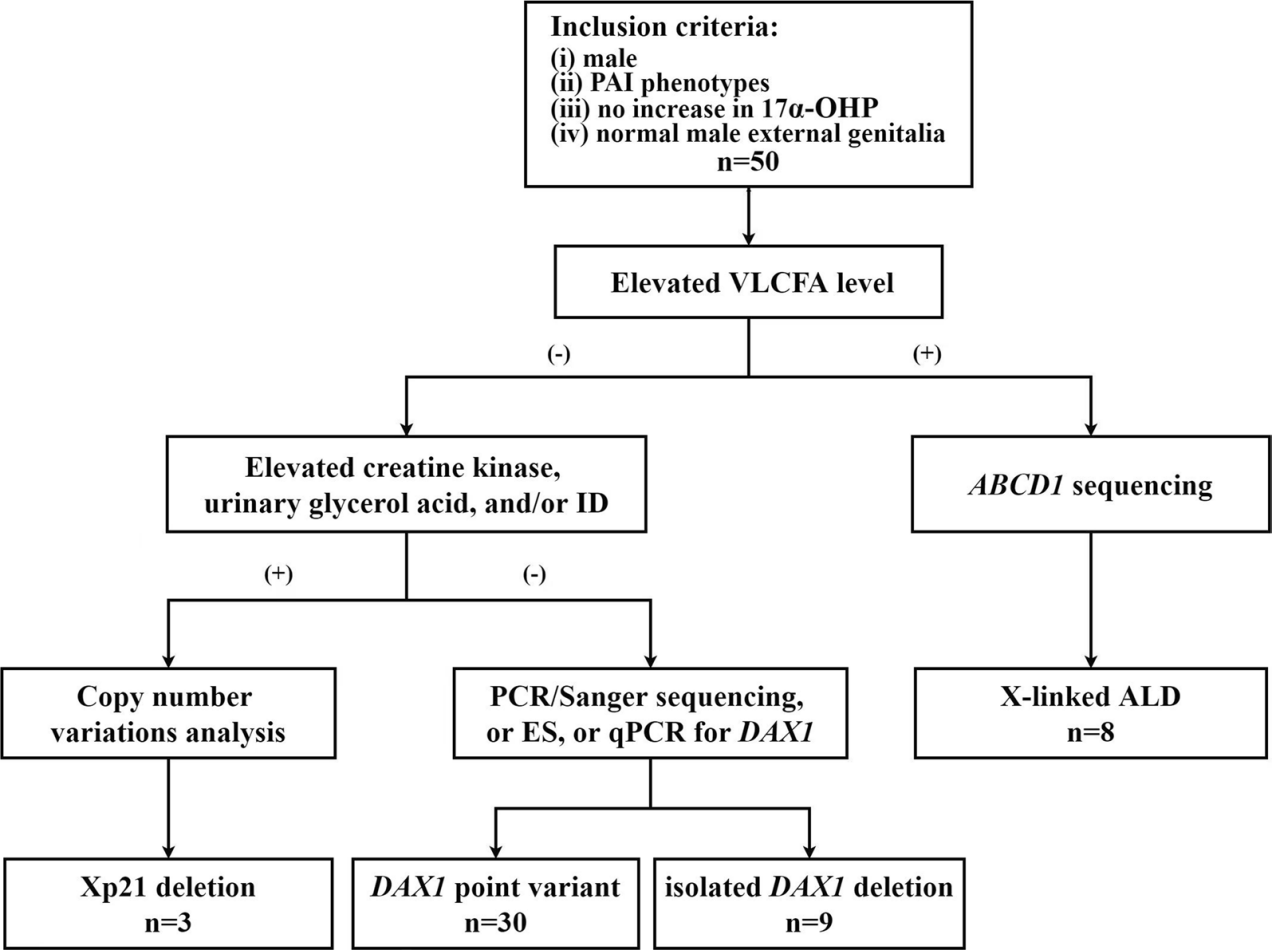
Diagnostic flowchart for X-linked AHC. *PAI* primary adrenal insufficiency; *17α-OHP* 17α-hydroxyprogesterone; *VLCFA* very-long-chain fatty acids; *ID* intellectual disability; *X-linked ALD* X-linked adrenoleukodystrophy; *PCR* polymerase chain reaction; *ES* exome sequencing; *qPCR* real-time quantitative PCR

Patients without acquired PAI (neoplasm, haemorrhage, infection, infiltration, and autoimmune disease) from Xinhua Hospital between February 2005 and April 2022 who met the following criteria were initially included in the study: (i) male; (ii) PAI phenotypes (hyperpigmentation, vomiting, increased ACTH, and decreased cortisol); (iii) no increase in 17α-hydroxyprogesterone (17α-OHP); and (iv) normal male external genitalia. A total of 50 paediatric male patients with a clinical diagnosis of PAI were included. These patients were evaluated and diagnosed by the following flowchart (Fig. 1). Very-long-chain fatty acid (VLCFA) analysis was performed first, and a total of eight patients exhibited increased VLCFA. These eight patients were excluded since X-linked ALD was further confirmed by genetic analysis. Then, the remaining 42 patients underwent a laboratory examination of creatine kinase and urinary glycerol acid levels, as well as an intellectual development assessment. If at least one of the above three items was abnormal, a chromosomal microarray (CMA) was then performed; otherwise, PCR/Sanger sequencing, exome sequencing (ES), or real-time quantitative polymerase chain reaction (qPCR) was applied. The study was approved by the ethics committee of the Xinhua Hospital, Shanghai Jiao Tong University School of Medicine (XHEC-WJW-2019-045). Written informed consent was obtained from patients or their guardians.

### Study design

A total of 42 eligible subjects from 39 unrelated families were divided into 39 cases with isolated DAX1 defects and three cases with Xp21 deletions. The following data were collected retrospectively: (i) demographic data; (ii) family history; (iii) age of onset and diagnosis; (iv) clinical features and laboratory findings at presentation and the last follow-up; (v) pubertal development of patients over 14 years of age; (vi) treatments for PAI and HH; and (vii) genetic results in the *DAX1* gene. The growth of the children was evaluated using the height standard deviation score (SDS) according to the Chinese child growth standards (2009 edition) [25]. The laboratory information included plasma ACTH levels (reference range: 5–60 pg/mL), cortisol levels (reference range: 185–624 nmol/L), serum sodium levels (Na^+^; reference range: 135–146 mmol/L), serum potassium levels (K^+^; reference range: 3.5–5.1 mmol/L), creatine kinase levels (reference range: 39–308 IU/L), urinary glycerol acid levels measured by gas chromatography–mass spectrometry (GC–MS) (reference range: 0.0–20.0 mmol/mol creatinine), FSH levels (reference range: 14 years, 0.45–10.46 IU/L; 15 years, 0.43–18.54 IU/L; 16 years, 0.16–9.65 IU/L; 17–19 years, 2.12–14.15 IU/L), LH levels (reference range: 14 years, 0.48–7.93 IU/L; 15 years, 0.50–10.73 IU/L; 16 years, 0.48–10.83 IU/L; 17–19 years, 1.20–5.49 IU/L), and testosterone levels (reference range: 13–15 years, 0.1–17.6 nmol/L; 16–18 years, 4.0–24.0 nmol/L) [26]. In the GnRH stimulation test, LH and FSH levels were measured at 0, 30, 60, 90, and 120 min after a single dose of 100 μg of intravenous GnRH. The normal pubertal response to the GnRH stimulation test is a peak value of LH of 8.0 IU/L. In hCG therapy, 1000–2000 IU of hCG was intramuscularly injected twice weekly. In the pulsatile GnRH administration, 10 μg of intravenous gonadorelin was administered every 1.5 h using a computerized portable infusion pump. VLCFA were evaluated through GC–MS using stable isotope dilution for quantification (C22:0, C24:0, C26:0, C26:0/C22:0, and C24:0/C22:0).

### Molecular analysis

Genomic DNA was extracted from peripheral blood leukocytes obtained from the patients and their parents using the QIAamp DNA Blood Mini Kit (QIAGEN, Valencia, CA, USA). Genetic tests were performed in 42 patients. Of those tests, 25/42 (60%) were polymerase chain reaction (PCR) and Sanger sequencing, 9/42 (21%) were PCR and real-time quantitative PCR (qPCR), 5/42 (12%) were exome sequencing (ES), and the remaining three (7%) were chromosomal microarray (CMA).

The genetic tests procedures were as follows: (i) Sanger sequencing: the entire coding regions and splice sites of the *DAX1* gene were amplified by PCR using the appropriate primers designed by PrimerPremier 5. Primer sequences are available upon request. The PCR products were sequenced by an automated sequencer (ABI PRISM 3730 Genetic Analyser; Applied Biosystems, Foster City, California, United States). The obtained sequences were analysed with Sequencing Analysis software version 5 (Life Technologies). (ii) qPCR: If no *DAX1* amplification was obtained by PCR, which indicated a gross deletion involving the *DAX1* gene, qPCR with SYBR Green I detection was used to confirm the gross deletion. Primer sequences for two coding exons are available upon request. The *ALB* gene was utilized as a reference gene for the relative quantification of target genes. (iii) ES: ES was performed using the capture kit of xGen Exome Research Panel (Integrated DNA Technologies, Coralville, IO, USA) as previously reported [27]. The variants at a frequency over 1% in the 1000 Genomes Project, Genome Aggregation Database (GnomAD), and Exome Variant Server (EVS) Database or at a frequency over 5% in the local database (containing approximately 6000 exomes) were excluded from the list of candidate variants. Then, we screened the selected variants for autosomal recessive inheritance, autosomal dominant/de novo, and X-linked inheritance patterns. The variants were finally classified following the guidelines of the American College of Medical Genetics and Genomics (ACMG). Sanger sequencing was further performed to confirm the variants detected by ES and identify their parental origins. (iv) CMA: One patient was analysed using Agilent 4 × 44 K (Santa Clara, California, USA). Two patients were analysed with the Affymetrix CytoScan™ 750 k (Santa Clara, California, USA). The chromosomal microarray experiment was performed using standardized protocols provided by the manufacturer. Affymetrix® Chromosome Analysis Suite (ChAS) 1.2.2 (Affymetrix Inc.) and Genomic Workbench software (Agilent, Inc.) were used to detect and analyse the chromosomal copy number variations (CNVs) identified in the patients. The chromosome positions are shown according to GRCh 37 (hg19).

### Statistical analysis

Statistical analysis was executed by GraphPad Prism version 8.0.1 for Windows (GraphPad Software Inc. San Diego, CA, USA; www.graphpad.com). Continuous variables that met the conditions of normal distribution and equal variance are presented as the means ± standard deviations; otherwise, they are shown as medians (interquartile ranges). The age of onset and diagnosis is shown as the median (range: min–max). Categorical variables are expressed as frequency distributions. The unpaired Student’s t test or Mann–Whitney U test was used to compare serum sodium, potassium and cortisol levels, and the χ2 test or Fisher's exact test was performed to compare the frequency of all the signs/symptoms in the different onset groups or groups carrying different *DAX1* variants. The unpaired Student’s t test or Mann–Whitney U test was applied in different pubertal development groups or different treatment groups, and the paired Student’s t test or Wilcoxon test was used before and after the intervention to compare the testicular volume and hormone levels. The level of significance was set at 0.05 (two-tailed).

## Results

### Clinical characteristics and treatment of PAI

All 42 patients with X-linked AHC from 39 non-consanguineous families were male, including 39 with isolated DAX1 defects from 36 unrelated families and three with Xp21 deletions. They were from 13 provinces across China (Table 1). Of the 42 patients, the median age of onset and of diagnosis was 1.0 month (range: 0.0–98.0) and 3.0 years (range: 0.1–15.0), respectively. The duration of the follow-up was 7.7 ± 4.5 years, and the age of the patient with the last follow-up was 9.0 ± 5.0 years old.

**Table 1:** Clinical features and genetic data of 42 patients with X-linked AHC

Patient ID	Age of Onset	Na^+^ (mmol/L)	K^+^ (mmol/L)	17α-OHP (nmol/L)	ACTH (pg/ml)	Cortisol (noml/L)	Family origin	Variants		ACMG classification	Diagnostic methods
								Nucleotide alteration NM_000475.4	Amino acid alteration		
1	25d	106	5.8	4.0	147	20	Hunan	c.252_255delinsAC*	p.T85Qfs*27	P	PCR + Sanger
2	2d	139	5.2	2.3	> 1250	7	Jiangsu	c.291del	p.E98Rfs*166	P	PCR + Sanger
3	5y	123	4.2	1.9	> 1250	159	Jiangsu	c.297del*	p.T100Rfs*164	P	PCR + Sanger
4	At birth	127	8.4	2.0	> 2000	154	Anhui	c.332_333dup*	p.D112Lfs*153	LP	ES + Sanger
5A	1y	118	4.1	< 0.2	> 1250	166	Jiangxi	c.501del	p.G169Afs*95	P	PCR + Sanger
5B	3.3y	136	5.7	< 0.2	1008	12	Jiangxi	c.501del	p.G169Afs*95	P	PCR + Sanger
6	At birth	116	9.3	2.0	768	14	Anhui	c.543dup*	p.G182Rfs*3	P	PCR + Sanger
7	7d	134	4.8	0.9	1188	286	Hubei	c.604del	p.C202Afs*62	LP	ES + Sanger
8A	5d	120	6.1	< 0.2	> 1250	99	Hubei	c.676del*	p.A226Lfs*38	P	PCR + Sanger
8B	3d	141	4.8	3.9	> 1250	23	Hubei	c.676del*	p.A226Lfs*38	P	PCR + Sanger
8C	3y	138	3.9	0.6	305	56	Hubei	c.676del*	p.A226Lfs*38	P	PCR + Sanger
9	7d	135	5.1	2.0	1932	134	Anhui	c.985del*	p.G330Afs*42	P	PCR + Sanger
10	6y	138	4	1.1	1542	12	Anhui	c.998_1001dup^#^	p.P335Tfs*55	P	PCR + Sanger
11	7y	130	5.3	1.4	> 2000	16	Anhui	c.998_1001dup^#^	p.P335Tfs*55	P	PCR + Sanger
12	8.2y	140	4.4	4.0	> 1250	39	Zhejiang	c.1034del*	p.P345Rfs*27	P	ES + Sanger
13	3d	134	5	2.9	> 2000	68	Shandong	c.1231_1234del	p.L411Vfs*6	P	PCR + Sanger
14	5.3y	124	5.8	2.3	510	400	Jiangxi	c.1292del	p.S431Ifs*6	P	PCR + Sanger
15	1 m	117	7.4	4.2	130	346	Zhejiang	c.1334del*	p.A445Vfs*17	LP	PCR + Sanger
16	14d	120	5.5	1.0	1375	37	Fujian	c.323C > A&*	p.S108*	P	ES + Sanger
17	7d	128	6.2	4.3	324	83	Jiangxi	c.528C > G	p.Y176*	P	PCR + Sanger
18	3.2y	127	4.6	0.5	> 2000	107	Fujian	c.535C > T*	p.Q179*	P	PCR + Sanger
19	6 m	138	4.3	< 0.2	1150	61	Zhejiang	c.685G > T*	p.E229*	P	PCR + Sanger
20	6 m	120	5.6	< 0.2	179	27	Shanghai	c.705G > A	p.W235*	P	PCR + Sanger
21	4.5y	128	6.1	< 0.2	> 2000	23	Zhejiang	c.1104C > A*	p.C368*	P	PCR + Sanger
22	At birth	115	7.2	1.2	> 1250	199	Zhejiang	c.833 T > C	p.L278P	P	ES + Sanger
23	10 m	126	7.3	1.9	> 2000	21	Fujian	c.884 T > C	p.L295P	LP	PCR + Sanger
24	At birth	142	5.3	1.5	> 2000	46	Jiangsu	c.1094 T > C	p.L365P	LP	PCR + Sanger
25	19d	137	4.3	2.3	119	11	Zhejiang	c.1157 T > G*	p.L386R	P	PCR + Sanger
26	1 m	130	5.5	< 0.2	> 1250	55	Shanxi	c.1157 T > C	p.L386P	LP	PCR + Sanger
27	11d	128	6.4	3.7	> 1250	19	Anhui	c.1275A > T*	p.R425S	LP	PCR + Sanger
28	1 m	125	8.2	1.1	390	159	Shanxi	exon 2 del	–	P	PCR + qPCR
29	3.5y	122	6.3	1.1	169	141	Heilongjiang	exon 2 del	–	P	PCR + qPCR
30	3d	117	6.9	4.0	291	487	Heilongjiang	exon 1–2 del	–	P	PCR + qPCR
31	13d	108	6.9	2.1	1290	19	Shanghai	exon 1–2 del	–	P	PCR + qPCR
32	15d	133	6.8	1.0	> 1250	65	Anhui	exon 1–2 del	–	P	PCR + qPCR
33	1 m	121	6.4	1.8	1986	156	Anhui	exon 1–2 del	–	P	PCR + qPCR
34	48d	129	5	1.7	> 1250	21	Anhui	exon 1–2 del	–	P	PCR + qPCR
35	50d	115	7.7	3.1	138	118	Henan	exon 1–2 del	–	P	PCR + qPCR
36	6 m	124	6.7	2.4	252	75	Jiangsu	exon 1–2 del	–	P	PCR + qPCR
37	3.3y	123	5.9	1.1	> 1250	109	Jiangsu	arr[hg19] Xp21.2p21.3(27,346,252–30,646,858) × 0	–	P	CMA
38	9d	124	8.9	2.0	> 2000	52	Guangxi	arr[hg19] Xp21.3p21.1(29,083,784–32,216,810) × 0	–	P	CMA
39	20d	124	8.2	2.1	77	133	Jiangxi	arr[hg19] Xp21.3p21.1(29,294,623–32,907,474) × 0	–	P	CMA
Reference range		135–146	3.5–5.1	< 6	5–60	185–624					

5A and 5B are cousins; 8A, 8B, and 8C are cousins

*D* day (s); *m* month(s); *y* year(s); &: de novo mutation; #: undetermined source; *: novel variants; P: pathogenic; LP: likely pathogenic; Sanger: Sanger sequencing; qPCR: real-time quantitative polymerase chain reaction; ES: exome sequencing; CMA: chromosomal microarray

P37 harboured *IL1RAPL1* and *DAX1* deletion; P38 and P39 carried the gross deletion including *IL1RAPL1*, *DAX1*, *GK*, and *DMD*

The most common initial feature was hyperpigmentation presenting in 90% (38/42) of the patients, followed by vomiting/diarrhoea in 48% (20/42), failure to thrive in 31% (13/42), and convulsions in 17% (7/42). Biochemical results showed increased ACTH levels [60% (25/42) over 1250 pg/ml] in all patients (42/42), decreased cortisol levels [63.1 (21.7–139.3) nmol/L] in 88% of patients (37/42), decreased serum sodium levels (126.7 ± 8.9 mmol/L) in 76% of patients (32/42) and increased serum potassium levels (6.0 ± 1.4 mmol/L) in 69% (29/42) of patients (Table 1).

An apparent bimodal distribution pattern for age at presentation was observed in 42 patients with X-linked AHC. Of those, 31 cases (74%) presented within one year of age with a median age of 15.0 days (range: 0.0–1.0 year), while 11 cases (26%) presented over three years of age with a median age of 4.5 years (range: 3.0–8.2). Therefore, 42 patients were divided into the infantile-onset group (≤ 1 year) and the childhood-onset group (> 1 year) according to the onset age (Table 2). There was no significant difference between these two groups in the frequency of all the signs/symptoms (all *P* > 0.05). In addition, a comparison of the biochemical parameters displayed no significant difference in the serum sodium and cortisol levels (both *P* > 0.05), but the serum potassium level of the infantile-onset group was higher than that of the childhood-onset group (6.3 ± 1.4 mmol/L vs. 5.1 ± 0.9 mmol/L, *P* = 0.0033). This finding indicated that the degree of electrolyte disturbance in infantile-onset patients was more severe than that in childhood-onset patients with X-linked AHC.

**Table 2:** Clinical and biochemical features of 42 patients with X-linked AHC

	Infantile-onset (n = 31)		Childhood-onset (n = 11)		*P* value
	n (%)	Value	n (%)	Value	
Hyperpigmentation	28 (90%)	–	10 (91%)	–	1.0000
Vomiting/diarrhoea	12 (39%)	–	8 (73%)	–	0.0523
Failure to thrive	11 (35%)	–	2 (18%)	–	0.4922
Convulsions	7 (23%)	–	0 (0%)	–	0.2093
Increased ACTH (pg/ml)	31 (100%)	–	11 (100%)	–	–
Decreased cortisol (nmol/L)	27 (87%)	65.1 (22.2–144.1)	10 (91%)	56.0 (19.3–125.0)	0.7565
Hyponatremia (mmol/L)	25 (81%)	125.6 ± 9.3	7 (64%)	129.9 ± 6.9	0.1182
Hyperkalaemia (mmol/L)	23 (74%)	6.3 ± 1.4	6 (55%)	5.1 ± 0.9	0.0033

Values are the means ± standard deviations or medians (interquartile ranges)

The hydrocortisone (HC) and 9α-fludrocortisone replacement therapy was a crucial treatment for adrenal dysfunction in all patients, and the doses were 13.3 ± 2.9 mg/m^2^/d and 0.05 (0.05–0.05) mg/d, respectively. Patients over 14 years of age (n = 13) had higher doses of HC than those under 14 years old (n = 29) (15.4 ± 2.7 mg/m^2^/d vs. 12.3 ± 2.5 mg/m^2^/d,* P* = 0.0027). After the treatment, all the patients showed remission in clinical signs and symptoms of PAI, hyperpigmentation disappeared gradually, and the serum electrolyte levels returned to normal, although the ACTH level [305.0 (54.3–539.0) pg/ml] remained mildly high.

### Evaluation of pubertal development and treatment for HH

Among the 42 patients, thirteen patients were over 14 years of age and were assessed for the conditions of pubertal development (Table 3). In these 13 patients, three exhibited spontaneous pubertal development, and ten encountered delayed puberty.

**Table 3:** Pubertal development in 13 patients and treatment for HH in seven patients

Patient ID	age (year)	Height SDS	Testes (ml)	FSH (IU/L)	LH (IU/L)	Testosterone (nmol/L)	GnRH stimulation test		Treatment duration (month)	Height SDS	Testes (ml)	FSH (IU/L)	LH (IU/L)	Testosterone (nmol/L)	GnRH stimulation test	
							Peak of FSH (IU/L)	Peak of							Peak of	Peak of
								LH							FSH	LH
								(IU/L)							(IU/L)	(IU/L)
**Pulsatile GnRH**																
16	14	0.43	3.5	1.58	< 0.20	< 0.35	4.99	0.59	1	0.38	4.5	6.57	1.77	1.43	6.32	1.94
20	14	− 1.50	3	4.12	0.23	< 0.35	6.27	1.35	3	0.19	4.5	8.37	0.95	1.12	9.31	0.99
32	14.5	− 1.67	3	2.82	0.39	< 0.35	1.93	1.69	4	− 0.58	9	3.63	3.42	4.56	3.34	0.91
**hCG (1000–2000 IU, twice weekly)**																
11	15.3	− 3.24	3	5.73	0.55	< 0.35	NE	NE	48	− 0.54	4	5.57	1.4	0.92		
14	14.1	− 2.31	2.5	1.52	0.2	< 0.35	1.54	0.64	5	− 2.29	3	1.46	0.62	1.39		
20	14	− 1.50	3	4.12	0.23	< 0.35	6.27	1.35	30	0.11	3.5	4.5	0.8	3.01		
23	14.2	− 2.76	2.5	1.5	< 0.20	< 0.35	2.18	0.21	6	− 1.88	3	0.81	< 0.20	1.14		
32	14.5	− 1.67	3	2.82	0.39	< 0.35	1.93	1.69	6	− 1.57	3	1.61	0.67	2.59		
34	14	− 1.71	2	1.25	0.13	0.62	1.41	0.16	7	− 1.39	3.5	0.99	0.1	14.86		
**Spontaneous pubertal development**																
2	12.8	− 1.05	6	9.21	1.17	5.79	–	–	–	–	–	–	–	–		
	16.9	− 1.54	3.5	7.75	0.87	3.92	9.19	3.66	–	–	–	–	–	–		
	17.3	− 0.40	3	4.77	0.42	0.49	NE	NE	NE	NE	NE	NE	NE	NE		
9	12.2	0.43	5	11.9	1.23	2.95	–	–	–	–	–	–	–	–		
	14.5	− 0.64	6	15.26	1.2	6.75	–	–	–	–	–	–	–	–		
10	12.6	− 1.14	5	10.38	0.53	3.06	–	–	–	–	–	–	–	–		
	15.5	− 0.65	8	10.21	3.24	4.73	–	–	–	–	–	–	–	–		
**Others**^**a**^																
3	14.1	− 2.35	2	2.45	0.24	< 0.35	1.77	< 0.20	NE	NE	NE	NE	NE	NE		
21	14	− 1.86	2.5	2.48	0.22	< 0.35	NE	NE	NE	NE	NE	NE	NE	NE		
37	14.2	− 1.89	2	2.68	0.24	< 0.35	NE	NE	NE	NE	NE	NE	NE	NE		

*hCG* human chorionic gonadotropin; *GnRH* gonadotropin-releasing hormone; *SDS* standard deviation score

^a^Patients without data on treatment outcomes; *NE* not evaluated; – not applicable

Reference range: FSH (follicle-stimulating hormone): 14 years, 0.45–10.46 IU/L; 15 years, 0.43–18.54 IU/L; 16 years, 0.16–9.65 IU/L; 17–19 years, 2.12–14.15 IU/L; LH (luteinizing hormone): 14 years, 0.48–7.93 IU/L; 15 years, 0.50–10.73 IU/L; 16 years, 0.48–10.83 IU/L; 17–19 years, 1.20–5.49 IU/L; testosterone: 13–15 years, 0.1–17.6 nmol/L; 16–18 years, 4.0–24.0 nmol/L; normal pubertal response to GnRH stimulation test: the peak value of LH by 8.0 IU/L

Spontaneous adolescent development was observed in three patients (P2, P9, and P10) during the follow-up at the age of 12.5 ± 0.3 years, with a testicular volume of 5.0 (5.0–5.5) ml, basal FSH level of 10.50 ± 1.35 IU/L, LH level of 0.98 ± 0.39 IU/L, and the testosterone level was 3.93 ± 1.61 nmol/L. The age of the last follow-up of these three patients was 15.6 ± 1.2 years old. Patient 2 was found to have pubertal arrest, with a decrease in testicular volume (from 6 to 3 ml) and testosterone level (from 5.79 nmol/L to 0.49 nmol/L) at 17.3 years old, suggesting arrested puberty. The peak LH and FSH levels after the GnRH stimulation test were 3.66 IU/L and 9.19 IU/L, respectively, suggesting the onset of HH. The other two patients (P9 and P10) had slow pubertal progression, and their testes were still around Tanner stage 2–3 (testicular volumes of 6 ml and 8 ml, respectively) two years after puberty initiation (Table 3). Ten patients with delayed puberty exhibited reduced testicular volume (2.6 ± 0.5 ml), low basal LH levels [0.23 (0.15–0.24) IU/L] and reduced testosterone production [< 0.35 nmol/L in all but one patient (P34, 0.62 nmol/L)] at 14.1 years of age (range: 14.0–15.3), suggesting the presence of HH. Of those patients, seven patients underwent the GnRH stimulation test, none of whom responded, with a peak FSH level of 1.93 (1.66–3.59) IU/L and a peak LH level of 0.59 (0.19–1.00) IU/L. These ten patients had significantly decreased testicular volume, basal FSH, LH, and testosterone levels compared to the three patients with spontaneous pubertal development (*P* = 0.0160, 0.0070, 0.0140, 0.0028, respectively). Therefore, in this cohort, eleven of the 13 patients over 14 years old had HH, including ten with delayed puberty and one with spontaneous pubertal development followed by disrupted puberty.

For the management of 11 patients with HH, four accepted hCG treatment (P11, P14, P23, and P34), and three received pulsatile GnRH administration (P16, P20, and P32). Of these three patients with pulsatile GnRH treatment, two patients (P20 and P32) had received hCG previously for 2.5 years and 6 months, respectively, but with limited effects. Thus, six patients had been treated with hCG. In addition, patient 2 recently underwent hCG therapy for less than one month, patient 21 received testosterone replacement, and the other two patients (P3 and P37) did not receive any management for HH. Therefore, a total of four patients (P2, P3, P21, and P37) had no data on treatment outcomes.

Six patients who received hCG therapy for 6.5 (6.0–24.3) months exhibited an enlargement of testicles from 2.7 ± 0.4 ml to 3.3 ± 0.4 ml (*P* = 0.0250). Their total testosterone levels increased from < 0.35 nmol/L in all but one patient (P34, 0.62 nmol/L) to 1.99 (1.20–2.91) nmol/L (*P* = 0.0313), which were still below the normal range for age in all patients except for one (P34, 14.86 nmol/L). The FSH and LH levels showed no change. This suggests that the increase in testicular volume and testosterone levels in the patients after hCG treatment was limited.

**Fig. 2:**
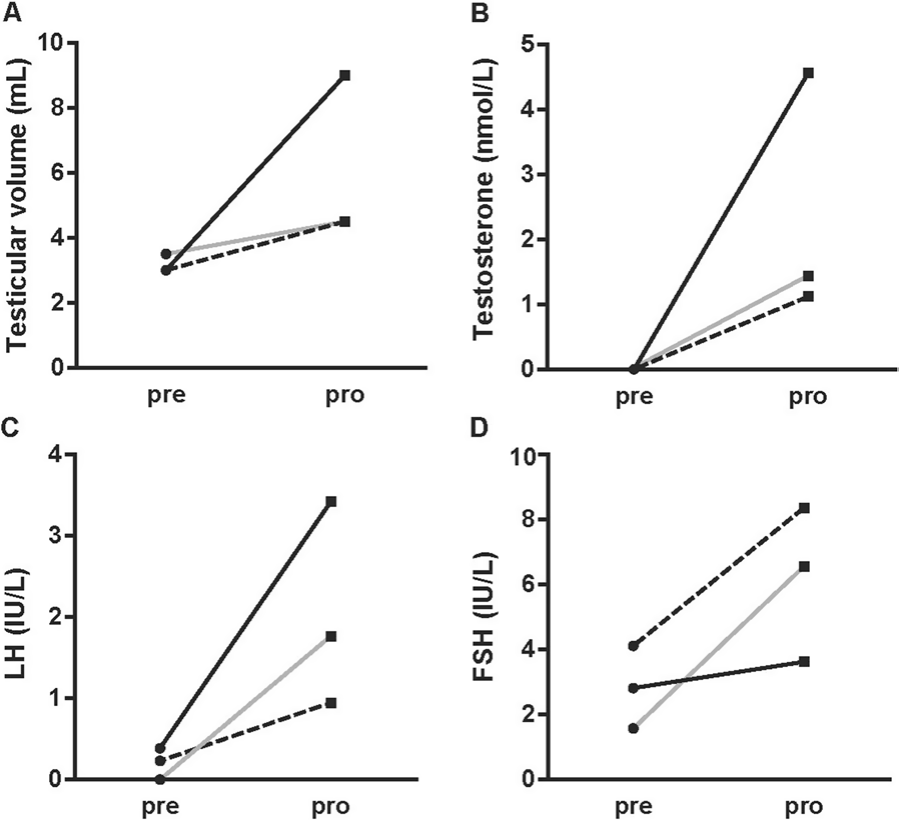
Treatment effects of pulsatile GnRH in three patients. Testicular volume (**A**), testosterone (**B**), LH (**C**), and FSH (**D**) changes before and after pulsatile GnRH treatment. *P16* solid grey line; *P20* black dotted line; *P32* solid black line

Three patients underwent pulsatile GnRH administration for 2.7 ± 1.5 months. A mild increase in testicular volumes [from 3.0 (3.0–3.3) ml to 4.5 (4.5–6.8) ml], basal LH levels [from 0.23 (0.12–0.31) IU/L to 1.77 (1.36–2.60) IU/L], basal FSH levels [from 2.84 ± 1.27 IU/L to 6.19 ± 2.39 IU/L], and testosterone levels [from all < 0.35 nmol/L to 1.43 (1.28–3.00) nmol/L] was observed (Fig. 2). There was still no response to the GnRH stimulation test in these three patients, with the peak LH level increasing from 1.21 ± 0.56 IU/L to 1.28 ± 0.57 IU/L and peak FSH level increasing from 4.40 ± 2.23 IU/L to 6.32 ± 2.99 IU/L. Thus, all three patients had some growth in terms of testicles, basal hormone levels, and GnRH-stimulated peak LH and FSH levels when comparing pre- and post-pulsatile GnRH treatment, although no significant difference was observed (all *P* > 0.05), probably due to the small sample size.

We compared the final testicular volume, LH, FSH, and testosterone levels between the six patients receiving hCG therapy at the last follow-up (n = 4) or prior to the therapy alteration (n = 2) and in three patients undergoing pulsatile GnRH administration at the last follow-up (n = 3). The testicles and LH levels of the patients undergoing pulsatile GnRH were larger and higher than those undergoing hCG therapy [4.5 (4.5–6.8) ml vs. 3.3 ± 0.4 ml, *P* = 0.0243] [1.77 (1.36–2.60) IU/L vs. 0.65 (0.23–0.77) IU/L, *P* = 0.0476]. Thus, pulsatile GnRH may be recommended for HH when hCG therapy is not satisfactory, although it is difficult to achieve normal testicular volume. Despite this, the height SDS of these 7 patients increased significantly after hCG medication and/or pulsatile GnRH therapy (− 1.82 ± 1.18 vs. − 0.87 ± 1.02, *P* = 0.0444), possibly due to the elevated testosterone levels after treatment.

### Genetic assessment

1. Xp21 deletion syndrome

Three patients (P37, P38 and P39) with Xp21 deletion syndrome were diagnosed by chromosomal microarray assays (Table 1). P37 showed hyperpigmentation and vomiting at 3.3 years old. When he was five years of age, a detailed clinical and biochemical examination was performed, which indicated mild intellectual disability, normal urinary glyceric acid and creatine kinase levels and adrenal dysfunction. Therefore, he was considered to have Xp21 deletion syndrome and diagnosed with *IL1RAPL1* and *DAX1* deletions (3.3 Mb) by CMA. He is currently 14.2 years of age, affected by intellectual disability and HH in addition to adrenal failure.

P38 presented with hyperpigmentation after birth and failure to thrive during the neonatal period. When he was six months of age, he manifested with delayed milestones (unable to lift his head) and hypotonia coupled with adrenal insufficiency. His two elder brothers had died at the ages of two months and four months. The laboratory findings showed that urinary glyceric acid level was elevated and creatine kinase in the blood was 5645 IU/L (reference range: 39–308 IU/L). Thus, Xp21 deletion syndrome was suspected by these clinical findings, and he was further diagnosed with *IL1RAPL1*, *DAX1*, *GK*, and *DMD* deletions (3.1 Mb) through CMA. Unfortunately, he died at the age of six months due to an adrenal crisis.

P39 encountered hyperpigmentation after birth. When he was two years old, he presented with failure to thrive and hypotonia, with a length of 80 cm (< − 2SD) and a weight of 8.8 kg (< − 2SD). Biochemical findings showed that the serum creatine kinase level was 9431 U/L and that the urinary glyceric acid level was 653.16 (reference range: 0.0–20.0 mmol/mol creatinine). His developmental quotient (DQ) scores in the domains of adaptive behaviour, gross motor, fine motor, language, and social behaviour assessed by the Gesell Development Diagnosis Scale were 23, 23, 25, 35, and 37, respectively, suggesting severe developmental delay. He was further diagnosed definitively with *IL1RAPL1*, *DAX1*, *GK*, and *DMD* (3.6 Mb) deletions by CMA. He is currently four years old, speaks few words and is unable to walk due to intellectual disability and persistent hypotonia.

2. DAX1 variant spectrum

**Fig. 3:**
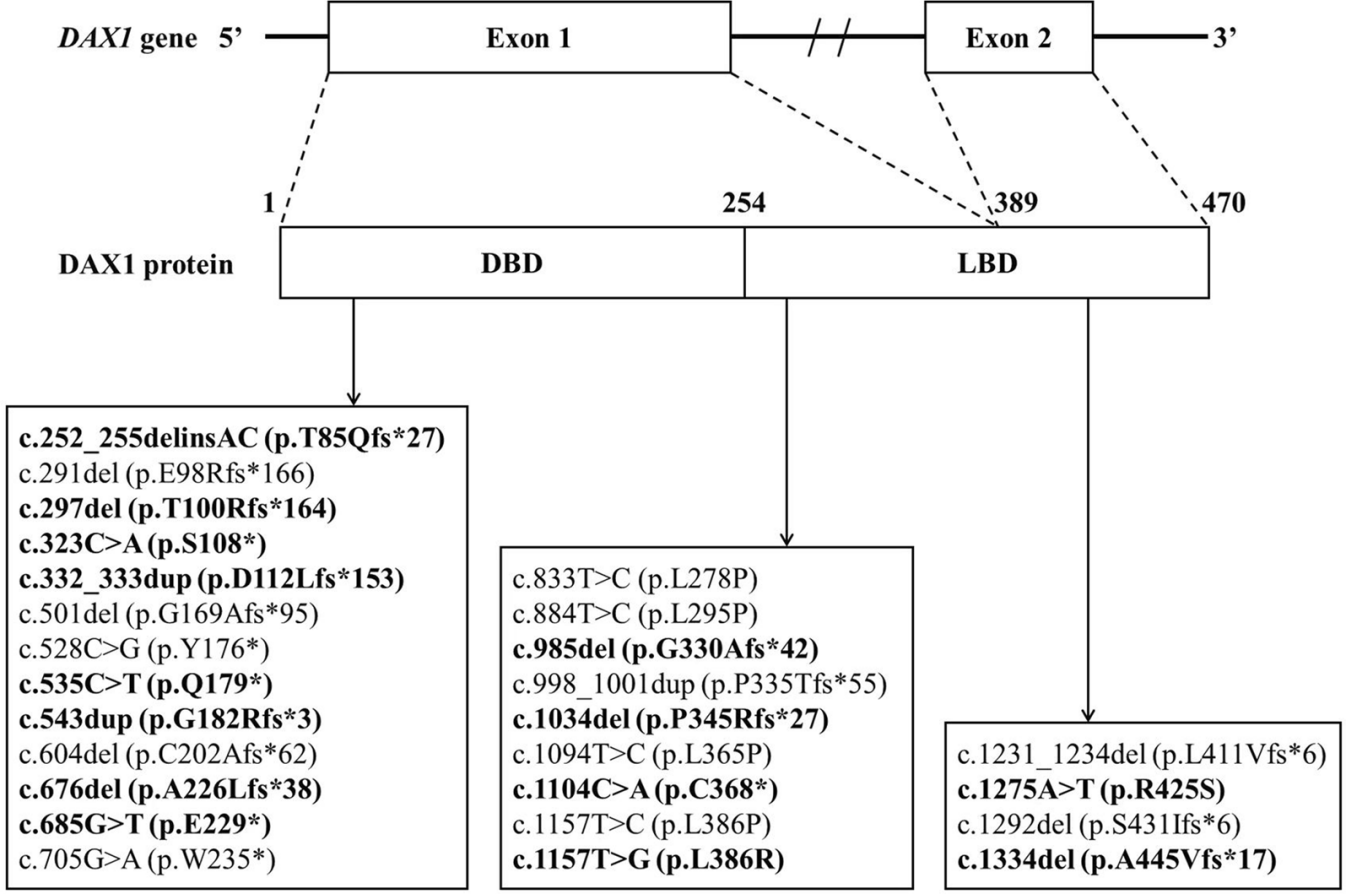
Twenty-six identified *DAX1* variants (frameshift, missense, and nonsense variants) in 30 patients. *DBD* DNA-binding domain; *LBD* putative ligand-binding domain; novel variants are in bold

A total of 30 variants in the *DAX1* gene were identified in 42 patients, including missense (n = 6), nonsense (n = 6), frameshift (n = 14), and gross deletions (n = 4), which were all classified as pathogenic (P) or likely pathogenic (LP) (Table 1, Fig. 3). Of the 30 *DAX1* variants, the entire *DAX1* deletion was the most frequent variant (10/42, 23.8%). Fourteen variants were not reported previously [28]. Frameshift and nonsense variants were distributed throughout the *DAX1* gene, located at both the DBD and LBD domains, whereas missense variants clustered in the LBD domain (Fig. 3).

Of the 42 patients, 18 had frameshift variants, six had nonsense changes, and six had missense changes. The remainder (n = 12, 29%) had a deletion of the locus containing *DAX1*. Seven of these deletions involved the entire *DAX1* gene, and two large deletions contained only exon 2 of *DAX1*, whereas three patients (P37, P38, and P39) harboured a contiguous gene deletion. Among these three patients, one (P37) had a deletion of *IL1RAPL1* and *DAX1*, and two (P38 and P39) carried the deletion extending distally to include the *IL1RAPL1*, *DAX1*, *GK*, and *DMD* loci.

3. Genotype–phenotype analysis

First, nine patients with a deletion involving *DAX1* alone presented with isolated X-linked AHC, while two patients (P38 and P39) at risk for the metabolic crisis or phenotypic characteristics of hypotonia and ID were found to have the deletion extended to contain the *IL1RAPL1*, *DAX1*, *GK* and *DMD* genes. A deletion extending to *IL1RAPL1* and *DAX1* was confirmed in one patient (P37) exhibiting ID in addition to adrenal dysfunction. Thus, a good genotype–phenotype correlation was confirmed in Xp21 deletion syndrome.

Then, an attempt was made to associate the severity of the disorder with specific variants. The age of onset of patients with X-linked AHC was used to assess the severity of adrenal insufficiency since it is the most readily available proxy. Of seven patients with isolated entire *DAX1* deletion and three with Xp21 deletion, all except for one patient (P37) had an early onset age of less than one year, indicating that the entire *DAX1* deletion might be associated with early onset age. In addition, no relation was found between the type of *DAX1* variant and whether pubertal development can be initiated spontaneously.

Finally, patients within a pedigree carrying the same variant may have different ages of onset. Patient 5A with the c.501del variant presented at one year of age, while his younger cousin (P5B) presented at 3.3 years of age. The age of onset of patient 8C with the c.676del variant was three years, while the age of onset of his younger cousins (P8A and P8B) was within the first five days and three days of life, respectively. It elucidated the phenotypic heterogeneity of X-linked AHC. In terms of phenotypic heterogeneity, genetic changes, epistatic interactions, and additional modifier variants may influence disease presentation in some single gene disorders. Although a group of modifier variants may influence the phenotype, not every modifier variant will be involved in every patient, implying a role for oligogenic inheritance in phenotypic expression [29, 30].

## Discussion

X-linked AHC is a rare congenital disorder, but it still accounts for a substantial proportion of patients with primary adrenal insufficiency. To date, few studies have been reported on large cohorts of X-linked AHC patients. Previous studies mainly focused on describing the genetic composition without detailed clinical characteristics of X-linked AHC [31–34]. The phenotype of X-linked AHC is heterogeneous, which causes a great challenge to clinical practice. In this study, we reported the first and largest cohort of 42 Chinese patients with X-linked AHC and analysed their clinical, biochemical, genetic, therapeutic, and follow-up characteristics.

The proportion of DAX1 defects in PAI is currently unclear. Of the cases with PAI followed up in our centre over 20 years, 52.5% (42/80) of patients with biochemically uncharacterized PAI carried DAX1 defects (unpublished data). The biochemically uncharacterized PAI is PAI that cannot be diagnosed by specific biochemical findings or urine/serum steroid metabolites (elevated 17α-OHP, elevated VLCFA, etc.), excluding congenital adrenal hyperplasia, X-linked ALD, and autoimmune disease [31, 32]. According to this criterion, studies from Japan, Italy, Turkey, and the United Kingdom showed that the proportion of DAX1 defects was 37.0% (20/54), 29.6% (16/54), 15.6% (12/77), and 11.7% (12/103), respectively, suggesting that DAX1 defects are a common cause of PAI [31–34]. Possible reasons for the different proportions of DAX1 defects in the above cohorts included ethnic differences, selection bias in enrolled patients, a small number of cases, and the different genetic compositions of PAI.

In DAX1, at least 200 distinct variants have been listed. The most prevalent variant in this study was the entire *DAX1* deletion (10/42, 23.8%), including isolated *DAX1* deletion (n = 7) and Xp21 deletion (n = 3). We compared the recurrent *DAX1* variants in the Italian, British, and Japanese cohorts and found that the entire *DAX1* deletion accounted for 25.0% (4/16), 16.7% (2/12), and 10.0% (2/20), respectively [31–34]. This suggests that the entire *DAX1* deletion may be the hotspot variant for X-linked AHC in these populations, accounting for approximately 10–25%. Furthermore, we compared the proportion of Xp21 deletion in X-linked AHC, which accounted for 25.0% (4/16) in Italy, 16.7% (2/12) in the United Kingdom, 7.1% (3/42) in our cohort, and 5.0% (1/20) in Japan [32–34]. Of the ten cases with Xp21 deletion mentioned above, six involved *DAX1*, *GK*, and *DMD*, three extended to include *IL1RAPL1*, *DAX1*, *GK*, and *DMD* (including our two patients), and one contained the locus of *IL1RAPL1* and *DAX1* (our patient). This suggests that most of the contiguous gene deletions in patients with DAX1-related X-linked AHC contain *DAX1*, *GK*, and *DMD* deletions. Therefore, we recommend that males with biochemically uncharacteristic PAI be first evaluated for serum creatine kinase, urinary glycerol acid, and mental development, followed by CNVs analysis to confirm the Xp21 deletion. In addition, the number of cases with Xp21 deletion in this study is small, and the long-term prognosis, including the progression of clinical features and the efficacy of comprehensive treatment, is still unknown. In our study, 26 *DAX1* point variants were found in 27 non-consanguineous families, with only p.P335Tfs*55 discovered in two unrelated families. In reviewing genetic data from Turkish, Italian, British, and Japanese cohorts [31–34], a few *DAX1* point variants were identified to occur in no more than two families (p.W235* in P20 of our study and one family of Turkish study, p.F364C and p.F449Sfs*13 in two families respectively of Italian study), suggesting the occurrence of the recurrence of *DAX1* point variants in unrelated families is a rare phenomenon [35].

The age of onset exhibits a bimodal distribution in X-linked AHC patients. The majority of patients (70–80%) presented in infancy, and the remainder presented insidiously later in childhood with an age range of two to nine years old [3, 7]. Similarly, 74% of patients presented within one year of age, and 26% were over three years old (3.0–8.2 years) in our study. In the current study, nine of the 31 infantile-onset patients carried the entire *DAX1* deletion, and all but one of the ten patients harbouring the entire *DAX1* deletion presented within the first year of life, suggesting that total *DAX1* deletion may be linked to an earlier age of onset. Nevertheless, no difference was found in the frequency of other types of variants between the infantile-onset group and childhood-onset group, which was consistent with previous studies [7]. There may be several factors that confound the age of onset analysis. For example, symptoms may be nonspecific and may have existed for some time prior to diagnosis. Additionally, different degrees of PAI clinical manifestations would be brought on by environmental pressures such as infections and operations, which may not be compatible with the severity of adrenal insufficiency. Moreover, the infant period is a particularly vulnerable time for adrenal failure. We found that serum potassium levels in our infantile-onset patients were significantly higher than those in childhood-onset patients. The explanation is that the demand for mineralocorticoids is possibly greater in infancy. Thus, patients with X-linked AHC either develop signs and symptoms of PAI, such as electrolyte disturbances, early in life, or they do not occur until later in life when they encounter severe disease or other environmental stress. Furthermore, the age at onset of adrenal insufficiency can vary within the same family, suggesting that other modifying genes and epigenetic as well as nongenetic factors are possibly involved in phenotypic variability in X-linked AHC [7, 10].

Most boys with X-linked AHC manifest HH as delayed puberty, whereas some boys can enter puberty spontaneously and progress to approximately Tanner stage 3 (or a testicular volume of 6–8 ml) between the ages of 15 and 18 years [18, 19]. In our study, 3 of 13 patients experienced spontaneous puberty, suggesting that a small proportion of patients (23.1%) had normal puberty initiation. However, there was no relationship between the type of *DAX1* variant and whether pubertal development can be initiated spontaneously in our study. Spontaneous pubertal development is possible due to the presence of residual hypothalamic and pituitary function in these patients, making partial gonadotropin deficiency sufficient to allow spontaneous pubertal development up to mid-puberty [18]. Disruption of puberty occurred in one of these three patients, and the other two had slow pubertal progression. The cessation of puberty may be due to the progressive degeneration of the hypothalamic-pituitary–gonadal axis of the X-linked AHC with age [14, 19]. Therefore, even if these patients have spontaneous pubertal development, their pubertal progression should be continuously monitored, as HH might occur subsequently.

DAX1 is expressed in the adrenal cortex, hypothalamus, pituitary, and testes. Previous studies revealed that HH in patients with DAX1 defects results from the combined deficiency of the hypothalamus and pituitary [24, 36, 37]. Nevertheless, it remains controversial which of these two levels is more seriously affected. The three patients in this study who accepted pulsatile GnRH demonstrated some growth in terms of LH, FSH, and testosterone, and their testicular volumes were larger than those of the six patients receiving hCG therapy (P < 0.05). This suggests that hypothalamic dysfunction in HH associated with DAX1 deficiency predominates. Furthermore, the treatment outcomes of pulsatile GnRH may not be as effective as in selective hypothalamic GnRH deficiency (e.g., Kallmann syndrome), suggesting at least a partial defect in the pituitary component, which results in impaired gonadotropin production [38–40]. Additionally, a slight response of testicular enlargement and rising testosterone was observed in six individuals treated with hCG medication; this response may not have been as strong as in patients who had their pituitary tumour resected, suggesting a primary Leydig cell defect in X-linked AHC [13, 23, 24]. Balsamo [41] has reported a case receiving gonadotropins (hCG and FSH) manifested testicular enlargement (6 ml), suggesting the potentially positive effect of FSH in the treatment for DAX1-related HH. The defect in DAX1 can impair the development and function of the adrenal cortex, hypothalamus, pituitary, and testicles, resulting in PAI, HH, and azoospermia. Due to the small number of HH cases and the insufficient follow-up time in this study, it remains uncertain which level in the hypothalamus or pituitary is more severely affected, making it unclear whether treatment with pulsatile GnRH or hCG is more effective in inducing puberty.

The evaluation of clinical features is the initial and important step in determining the possible aetiology of X-linked AHC. In the current study, we clarified the diagnosis of 42 patients with X-linked AHC based on biochemical characteristics (VLCFA, creatine kinase, and urinary glycerol acid) and intellectual development assessment, along with genetic analysis. As a result, we suggest a flowchart for diagnosing X-linked AHC in children (Fig. 1). First, the VLCFA level is a specific biomarker for the differential diagnosis of X-linked AHC and X-linked ALD. When males present with signs and symptoms of PAI along with normal VLCFA, X-linked AHC is suspected. Then, the differential diagnosis of isolated DAX1 defect and Xp21 deletion is subsequently made using extensive clinical and biochemical tests, such as creatine kinase, urinary glycerol acid, and intellectual performance assessment. If a patient shows an abnormality in any of these items, Xp21 deletion should be suspected, and CNVs analysis is needed for additional confirmation; otherwise, an isolated DAX1 defect should be taken into consideration. To confirm the *DAX1* variant, PCR/Sanger sequencing is performed. If PCR results show no *DAX1* amplification, indicating an isolated *DAX1* deletion, qPCR must be used to confirm the gross deletion. Diagnoses and differential diagnoses can be made more quickly and affordably using this flowchart.

## Conclusions

This study reported 42 patients with X-linked AHC, expanding our understanding of the clinical, biochemical, genetic, therapeutic, and follow-up characteristics of X-linked AHC. Patients with X-linked AHC exhibit a bimodal distribution of age of onset, with approximately 70% presenting within one year of age. The entire *DAX1* deletion appears to be related to an earlier age of onset. For pubertal development, the majority of boys with DAX1-related HH manifest with delayed puberty, while a small proportion of patients can spontaneously enter puberty followed by disruption. For the management of HH, all patients treated with either hCG or pulsatile GnRH had some degree of increase in testicular volumes and hormone concentrations but failed to reach normal levels. We propose a diagnostic process for children with X-linked AHC that will facilitate the rapid and comprehensive diagnosis of patients. A long-term follow-up and a larger sample size are needed to further evaluate prognosis and treatment.

## Data Availability

The datasets used and/or analysed in this current study are available from the corresponding author upon reasonable request.

## Abbreviations

AHC: Adrenal hypoplasia congenita
DAX1: Dosage-sensitive sex reversal-adrenal hypoplasia congenita critical region on X chromosome 1, gene 1
NR0B1: Nuclear receptor subfamily 0, Group B, member 1
DBD: DNA binding domain
LBD: Ligand-binding domain
HPG: Hypothalamic-pituitary–gonadal
PAI: Primary adrenal insufficiency
ACTH: Adrenocorticotropic hormone
HH: Hypogonadotropic hypogonadism
ID: Intellectual disability
SDS: Standard deviation score
17α-OHP: 17α-Hydroxyprogesterone
VLCFA: Very-long-chain fatty acids
ALD: Adrenoleukodystrophy
GC–MS: Gas chromatography–mass spectrometry
CMA: Chromosomal microarray
ES: Exome sequencing
qPCR: Real-time quantitative polymerase chain reaction
CNVs: Copy number variations
GnRH: Gonadotropin-releasing hormone
hCG: Human chorionic gonadotropin
FSH: Follicle-stimulating hormone
LH: Luteinizing hormone
HC: Hydrocortisone
